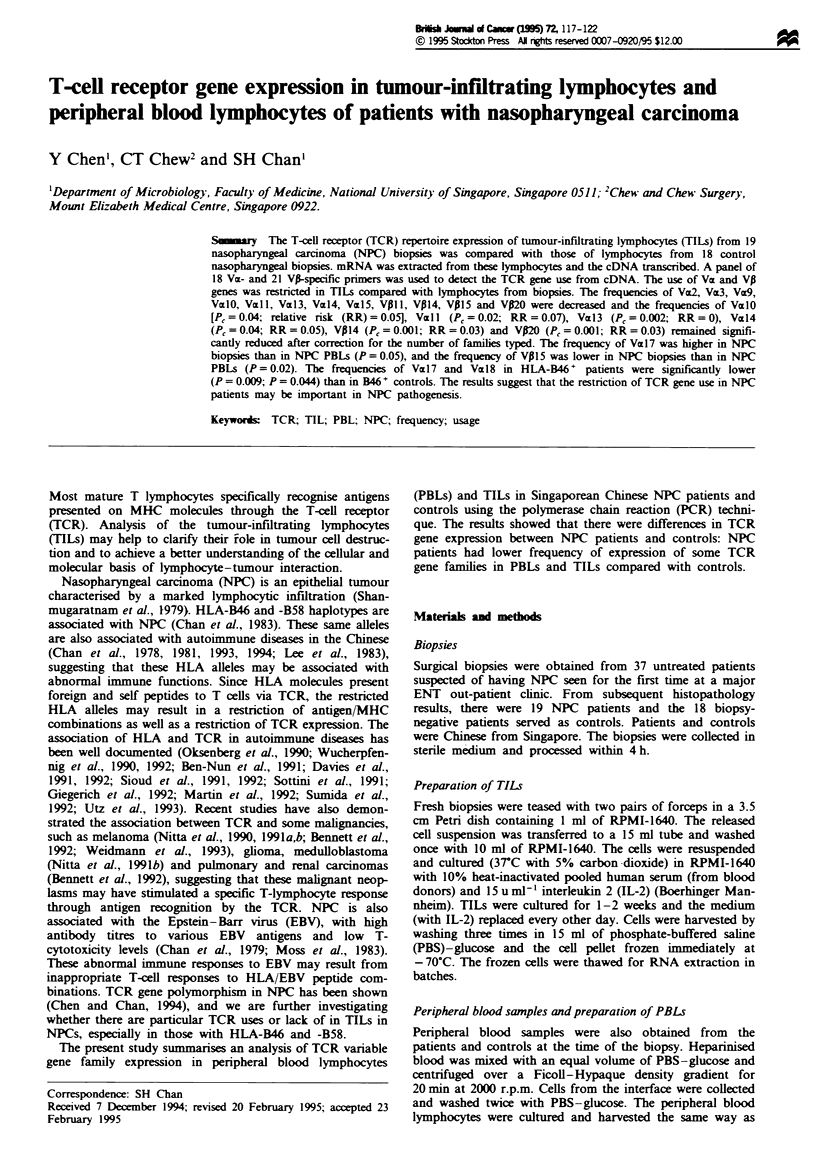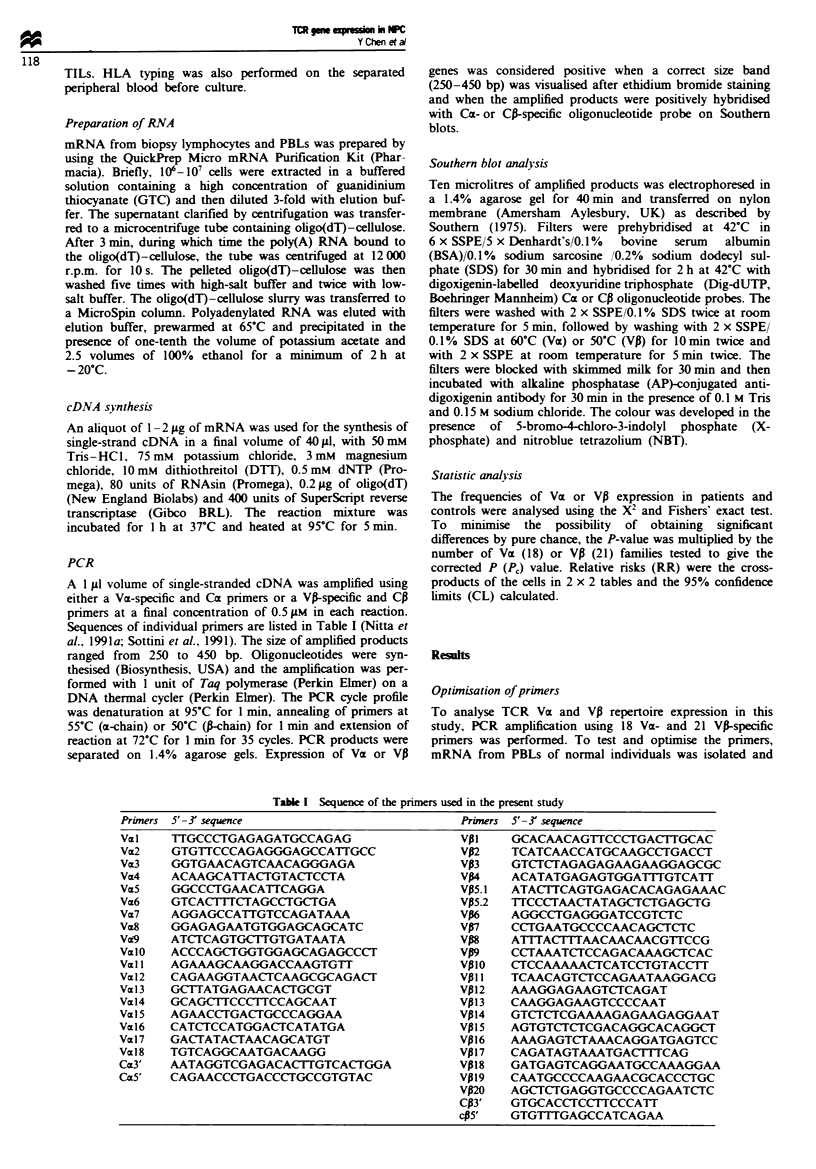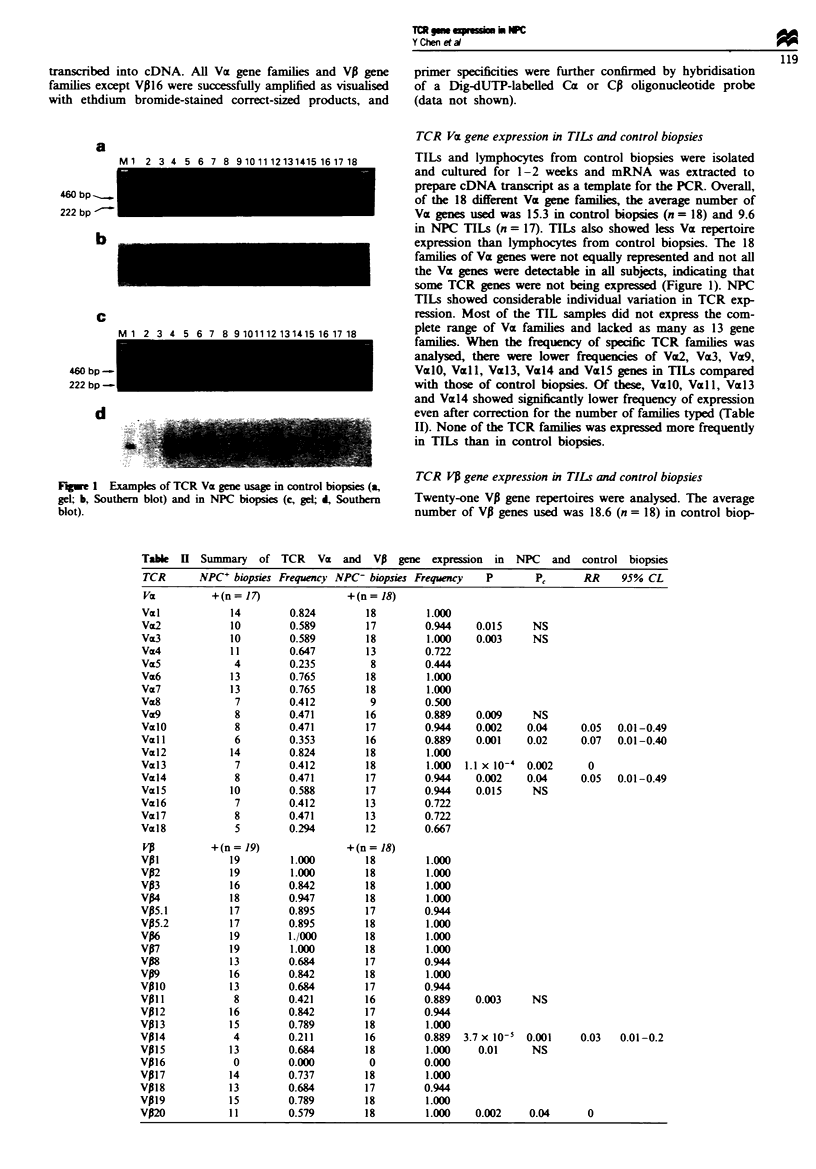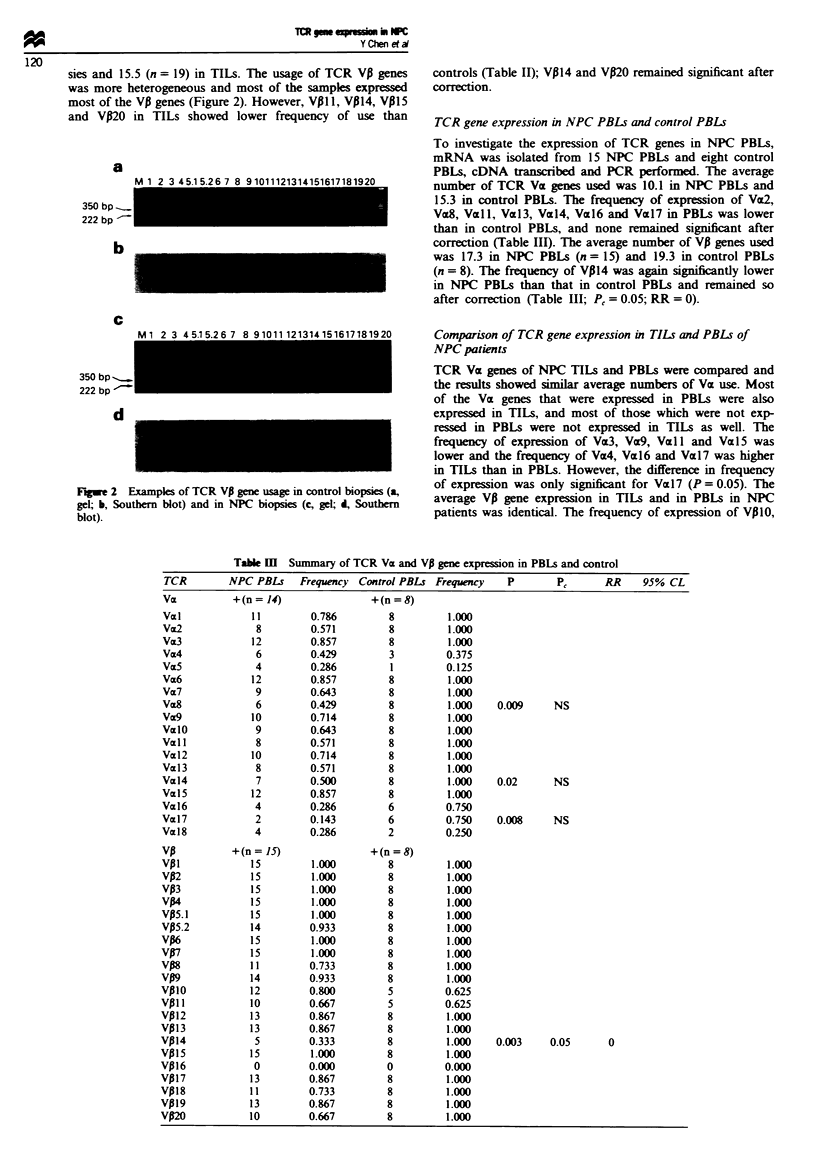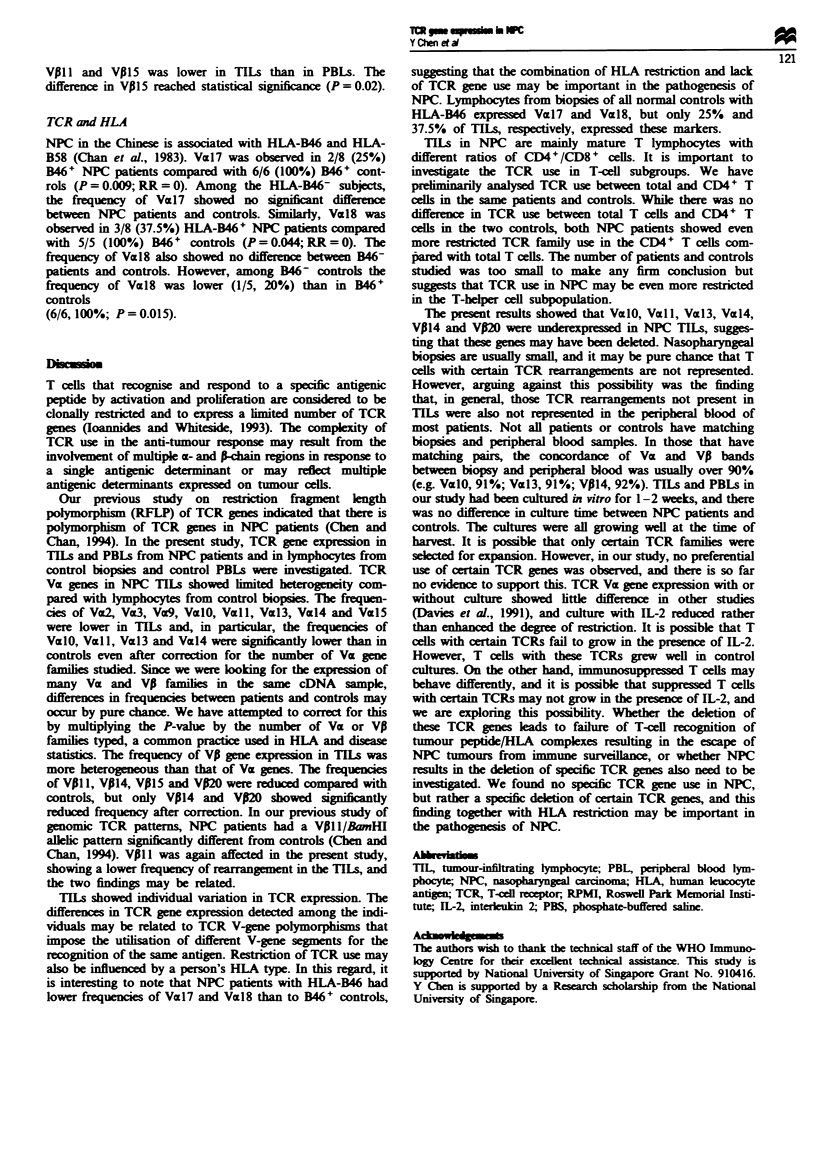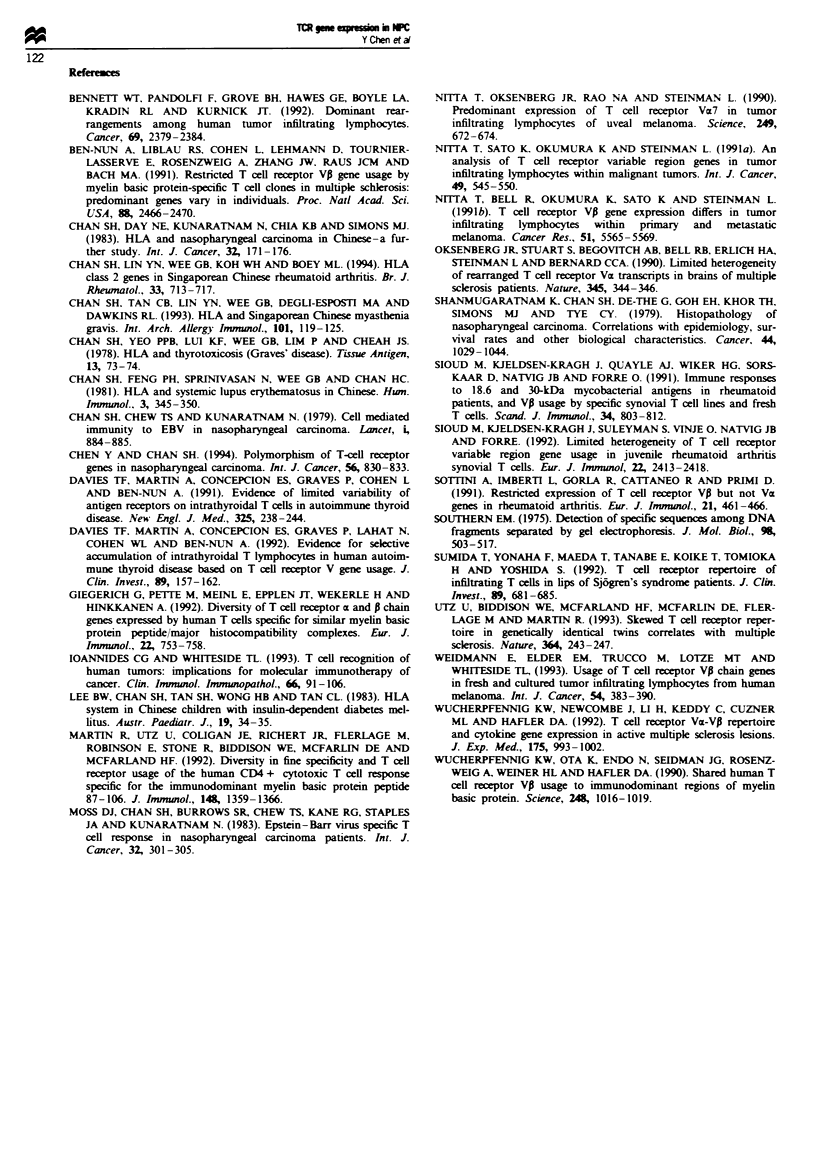# T-cell receptor gene expression in tumour-infiltrating lymphocytes and peripheral blood lymphocytes of patients with nasopharyngeal carcinoma.

**DOI:** 10.1038/bjc.1995.286

**Published:** 1995-07

**Authors:** Y. Chen, C. T. Chew, S. H. Chan

**Affiliations:** Department of Microbiology, Faculty of Medicine, National University of Singapore.

## Abstract

**Images:**


British Journal of Cancer (1995) 72, 117-122

© 1995 Stockton Press All rights reserved 0007-0920/95 $12.00

### T-cell receptor gene expression in tumour-infiltrating lymphocytes and peripheral blood lymphocytes of patients with nasopharyngeal carcinoma

Y Chen[1], CT Chew[2] and SH Chan[1]

[1]*Department of Microbiology, Faculty of Medicine, National University of Singapore, Singapore 0511;* [2]*Chew and Chew Surgery, Mount Elizabeth Medical Centre, Singapore 0922.*

**Summary** The T-cell receptor (TCR) repertoire expression of tumour-infiltrating lymphocytes (TILs) from 19 nasopharyngeal carcinoma (NPC) biopsies was compared with those of lymphocytes from 18 control nasopharyngeal biopsies. mRNA was extracted from these lymphocytes and the cDNA transcribed. A panel of 18 Vα- and 21 Vβ-specific primers was used to detect the TCR gene use from cDNA. The use of Vα and Vβ genes was restricted in TILs compared with lymphocytes from biopsies. The frequencies of Vα2, Vα3, Vα9, Vα10, Vα11, Vα13, Vα14, Vα15, Vβ11, Vβ14, Vβ15 and Vβ20 were decreased and the frequencies of Vα10 [$P_c = 0.04$; relative risk (RR) = 0.05], Vα11 ($P_c = 0.02$; RR = 0.07), Vα13 ($P_c = 0.002$; RR = 0), Vα14 ($P_c = 0.04$; RR = 0.05), Vβ14 ($P_c = 0.001$; RR = 0.03) and Vβ20 ($P_c = 0.001$; RR = 0.03) remained significantly reduced after correction for the number of families typed. The frequency of Vα17 was higher in NPC biopsies than in NPC PBLs ($P = 0.05$), and the frequency of Vβ15 was lower in NPC biopsies than in NPC PBLs ($P = 0.02$). The frequencies of Vα17 and Vα18 in HLA-B46$^+$ patients were significantly lower ($P = 0.009$; $P = 0.044$) than in B46$^+$ controls. The results suggest that the restriction of TCR gene use in NPC patients may be important in NPC pathogenesis.

**Keywords:** TCR; TIL; PBL; NPC; frequency; usage

Most mature T lymphocytes specifically recognise antigens presented on MHC molecules through the T-cell receptor (TCR). Analysis of the tumour-infiltrating lymphocytes (TILs) may help to clarify their role in tumour cell destruction and to achieve a better understanding of the cellular and molecular basis of lymphocyte-tumour interaction.

Nasopharyngeal carcinoma (NPC) is an epithelial tumour characterised by a marked lymphocytic infiltration (Shanmugaratnam *et al.*, 1979). HLA-B46 and -B58 haplotypes are associated with NPC (Chan *et al.*, 1983). These same alleles are also associated with autoimmune diseases in the Chinese (Chan *et al.*, 1978, 1981, 1993, 1994; Lee *et al.*, 1983), suggesting that these HLA alleles may be associated with abnormal immune functions. Since HLA molecules present foreign and self peptides to T cells via TCR, the restricted HLA alleles may result in a restriction of antigen/MHC combinations as well as a restriction of TCR expression. The association of HLA and TCR in autoimmune diseases has been well documented (Oksenberg *et al.*, 1990; Wucherpfennig *et al.*, 1990, 1992; Ben-Nun *et al.*, 1991; Davies *et al.*, 1991, 1992; Sioud *et al.*, 1991, 1992; Sottini *et al.*, 1991; Giegerich *et al.*, 1992; Martin *et al.*, 1992; Sumida *et al.*, 1992; Utz *et al.*, 1993). Recent studies have also demonstrated the association between TCR and some malignancies, such as melanoma (Nitta *et al.*, 1990, 1991*a,b*; Bennett *et al.*, 1992; Weidmann *et al.*, 1993), glioma, medulloblastoma (Nitta *et al.*, 1991*b*) and pulmonary and renal carcinomas (Bennett *et al.*, 1992), suggesting that these malignant neoplasms may have stimulated a specific T-lymphocyte response through antigen recognition by the TCR. NPC is also associated with the Epstein-Barr virus (EBV), with high antibody titres to various EBV antigens and low T-cytotoxicity levels (Chan *et al.*, 1979; Moss *et al.*, 1983). These abnormal immune responses to EBV may result from inappropriate T-cell responses to HLA/EBV peptide combinations. TCR gene polymorphism in NPC has been shown (Chen and Chan, 1994), and we are further investigating whether there are particular TCR uses or lack of in TILs in NPCs, especially in those with HLA-B46 and -B58.

The present study summarises an analysis of TCR variable gene family expression in peripheral blood lymphocytes (PBLs) and TILs in Singaporean Chinese NPC patients and controls using the polymerase chain reaction (PCR) technique. The results showed that there were differences in TCR gene expression between NPC patients and controls: NPC patients had lower frequency of expression of some TCR gene families in PBLs and TILs compared with controls.

Correspondence: SH Chan
Received 7 December 1994; revised 20 February 1995; accepted 23 February 1995

### Materials and methods

### *Biopsies*

Surgical biopsies were obtained from 37 untreated patients suspected of having NPC seen for the first time at a major ENT out-patient clinic. From subsequent histopathology results, there were 19 NPC patients and the 18 biopsy-negative patients served as controls. Patients and controls were Chinese from Singapore. The biopsies were collected in sterile medium and processed within 4 h.

### *Preparation of TILs*

Fresh biopsies were teased with two pairs of forceps in a 3.5 cm Petri dish containing 1 ml of RPMI-1640. The released cell suspension was transferred to a 15 ml tube and washed once with 10 ml of RPMI-1640. The cells were resuspended and cultured (37°C with 5% carbon dioxide) in RPMI-1640 with 10% heat-inactivated pooled human serum (from blood donors) and 15 u ml$^{-1}$ interleukin 2 (IL-2) (Boerhinger Manheim). TILs were cultured for 1-2 weeks and the medium (with IL-2) replaced every other day. Cells were harvested by washing three times in 15 ml of phosphate-buffered saline (PBS)-glucose and the cell pellet frozen immediately at −70°C. The frozen cells were thawed for RNA extraction in batches.

### *Peripheral blood samples and preparation of PBLs*

Peripheral blood samples were also obtained from the patients and controls at the time of the biopsy. Heparinised blood was mixed with an equal volume of PBS-glucose and centrifuged over a Ficoll-Hypaque density gradient for 20 min at 2000 r.p.m. Cells from the interface were collected and washed twice with PBS-glucose. The peripheral blood lymphocytes were cultured and harvested the same way as

TILs. HLA typing was also performed on the separated peripheral blood before culture.

*Preparation of RNA*

mRNA from biopsy lymphocytes and PBLs was prepared by using the QuickPrep Micro mRNA Purification Kit (Pharmacia). Briefly, $10^6-10^7$ cells were extracted in a buffered solution containing a high concentration of guanidinium thiocyanate (GTC) and then diluted 3-fold with elution buffer. The supernatant clarified by centrifugation was transferred to a microcentrifuge tube containing oligo(dT)-cellulose. After 3 min, during which time the poly(A) RNA bound to the oligo(dT)-cellulose, the tube was centrifuged at 12 000 r.p.m. for 10 s. The pelleted oligo(dT)-cellulose was then washed five times with high-salt buffer and twice with low-salt buffer. The oligo(dT)-cellulose slurry was transferred to a MicroSpin column. Polyadenylated RNA was eluted with elution buffer, prewarmed at 65°C and precipitated in the presence of one-tenth the volume of potassium acetate and 2.5 volumes of 100% ethanol for a minimum of 2 h at −20°C.

*cDNA synthesis*

An aliquot of 1–2 μg of mRNA was used for the synthesis of single-strand cDNA in a final volume of 40 μl, with 50 mM Tris-HCl, 75 mM potassium chloride, 3 mM magnesium chloride, 10 mM dithiothreitol (DTT), 0.5 mM dNTP (Promega), 80 units of RNAsin (Promega), 0.2 μg of oligo(dT) (New England Biolabs) and 400 units of SuperScript reverse transcriptase (Gibco BRL). The reaction mixture was incubated for 1 h at 37°C and heated at 95°C for 5 min.

*PCR*

A 1 μl volume of single-stranded cDNA was amplified using either a Vα-specific and Cα primers or a Vβ-specific and Cβ primers at a final concentration of 0.5 μM in each reaction. Sequences of individual primers are listed in Table I (Nitta *et al.*, 1991*a*; Sottini *et al.*, 1991). The size of amplified products ranged from 250 to 450 bp. Oligonucleotides were synthesised (Biosynthesis, USA) and the amplification was performed with 1 unit of *Taq* polymerase (Perkin Elmer) on a DNA thermal cycler (Perkin Elmer). The PCR cycle profile was denaturation at 95°C for 1 min, annealing of primers at 55°C (α-chain) or 50°C (β-chain) for 1 min and extension of reaction at 72°C for 1 min for 35 cycles. PCR products were separated on 1.4% agarose gels. Expression of Vα or Vβ genes was considered positive when a correct size band (250–450 bp) was visualised after ethidium bromide staining and when the amplified products were positively hybridised with Cα- or Cβ-specific oligonucleotide probe on Southern blots.

*Southern blot analysis*

Ten microlitres of amplified products was electrophoresed in a 1.4% agarose gel for 40 min and transferred on nylon membrane (Amersham Aylesbury, UK) as described by Southern (1975). Filters were prehybridised at 42°C in 6 × SSPE/5 × Denhardt's/0.1% bovine serum albumin (BSA)/0.1% sodium sarcosine /0.2% sodium dodecyl sulphate (SDS) for 30 min and hybridised for 2 h at 42°C with digoxigenin-labelled deoxyuridine triphosphate (Dig-dUTP, Boehringer Mannheim) Cα or Cβ oligonucleotide probes. The filters were washed with 2 × SSPE/0.1% SDS twice at room temperature for 5 min, followed by washing with 2 × SSPE/0.1% SDS at 60°C (Vα) or 50°C (Vβ) for 10 min twice and with 2 × SSPE at room temperature for 5 min twice. The filters were blocked with skimmed milk for 30 min and then incubated with alkaline phosphatase (AP)-conjugated anti-digoxigenin antibody for 30 min in the presence of 0.1 M Tris and 0.15 M sodium chloride. The colour was developed in the presence of 5-bromo-4-chloro-3-indolyl phosphate (X-phosphate) and nitroblue tetrazolium (NBT).

*Statistic analysis*

The frequencies of Vα or Vβ expression in patients and controls were analysed using the $X^2$ and Fishers' exact test. To minimise the possibility of obtaining significant differences by pure chance, the *P*-value was multiplied by the number of Vα (18) or Vβ (21) families tested to give the corrected *P* ($P_c$) value. Relative risks (RR) were the cross-products of the cells in 2 × 2 tables and the 95% confidence limits (CL) calculated.

### Results

*Optimisation of primers*

To analyse TCR Vα and Vβ repertoire expression in this study, PCR amplification using 18 Vα- and 21 Vβ-specific primers was performed. To test and optimise the primers, mRNA from PBLs of normal individuals was isolated and

**Table I** Sequence of the primers used in the present study

| *Primers* | *5′–3′ sequence* | *Primers* | *5′–3′ sequence* |
|---|---|---|---|
| Vα1 | TTGCCCTGAGAGATGCCAGAG | Vβ1 | GCACAACAGTTCCCTGACTTGCAC |
| Vα2 | GTGTTCCCAGAGGGAGCCATTGCC | Vβ2 | TCATCAACCATGCAAGCCTGACCT |
| Vα3 | GGTGAACAGTCAACAGGGAGA | Vβ3 | GTCTCTAGAGAGAAGAAGGAGCGC |
| Vα4 | ACAAGCATTACTGTACTCCTA | Vβ4 | ACATATGAGAGTGGATTTGTCATT |
| Vα5 | GGCCCTGAACATTCAGGA | Vβ5.1 | ATACTTCAGTGAGACACAGAGAAAC |
| Vα6 | GTCACTTTCTAGCCTGCTGA | Vβ5.2 | TTCCCTAACTATAGCTCTGAGCTG |
| Vα7 | AGGAGCCATTGTCCAGATAAA | Vβ6 | AGGCCTGAGGGATCCGTCTC |
| Vα8 | GGAGAGAATGTGGAGCAGCATC | Vβ7 | CCTGAATGCCCCAACAGCTCTC |
| Vα9 | ATCTCAGTGCTTGTGATAATA | Vβ8 | ATTTACTTTAACAACAACGTTCCG |
| Vα10 | ACCCAGCTGGTGGAGCAGAGCCCT | Vβ9 | CCTAAATCTCCAGACAAAGCTCAC |
| Vα11 | AGAAAGCAAGGACCAAGTGTT | Vβ10 | CTCCAAAAACTCATCCTGTACCTT |
| Vα12 | CAGAAGGTAACTCAAGCGCAGACT | Vβ11 | TCAACAGTCTCCAGAATAAGGACG |
| Vα13 | GCTTATGAGAACACTGCGT | Vβ12 | AAAGGAGAAGTCTCAGAT |
| Vα14 | GCAGCTTCCCTTCCAGCAAT | Vβ13 | CAAGGAGAAGTCCCCAAT |
| Vα15 | AGAACCTGACTGCCCAGGAA | Vβ14 | GTCTCTCGAAAAGAGAAGAGGAAT |
| Vα16 | CATCTCCATGGACTCATATGA | Vβ15 | AGTGTCTCTCGACAGGCACAGGCT |
| Vα17 | GACTATACTAACAGCATGT | Vβ16 | AAAGAGTCTAAACAGGATGAGTCC |
| Vα18 | TGTCAGGCAATGACAAGG | Vβ17 | CAGATAGTAAATGACTTTCAG |
| Cα3′ | AATAGGTCGAGACACTTGTCACTGGA | Vβ18 | GATGAGTCAGGAATGCCAAAGGAA |
| Cα5′ | CAGAACCCTGACCCTGCCGTGTAC | Vβ19 | CAATGCCCCAAGAACGCACCCTGC |
| | | Vβ20 | AGCTCTGAGGTGCCCCAGAATCTC |
| | | Cβ3′ | GTGCACCTCCTTCCCATT |
| | | cβ5′ | GTGTTTGAGCCATCAGAA |

transcribed into cDNA. All Vα gene families and Vβ gene families except Vβ16 were successfully amplified as visualised with ethdium bromide-stained correct-sized products, and primer specificities were further confirmed by hybridisation of a Dig-dUTP-labelled Cα or Cβ oligonucleotide probe (data not shown).

**Figure 1** Examples of TCR Vα gene usage in control biopsies (**a**, gel; **b**, Southern blot) and in NPC biopsies (**c**, gel; **d**, Southern blot).

### *TCR Vα gene expression in TILs and control biopsies*

TILs and lymphocytes from control biopsies were isolated and cultured for 1–2 weeks and mRNA was extracted to prepare cDNA transcript as a template for the PCR. Overall, of the 18 different Vα gene families, the average number of Vα genes used was 15.3 in control biopsies ($n = 18$) and 9.6 in NPC TILs ($n = 17$). TILs also showed less Vα repertoire expression than lymphocytes from control biopsies. The 18 families of Vα genes were not equally represented and not all the Vα genes were detectable in all subjects, indicating that some TCR genes were not being expressed (Figure 1). NPC TILs showed considerable individual variation in TCR expression. Most of the TIL samples did not express the complete range of Vα families and lacked as many as 13 gene families. When the frequency of specific TCR families was analysed, there were lower frequencies of Vα2, Vα3, Vα9, Vα10, Vα11, Vα13, Vα14 and Vα15 genes in TILs compared with those of control biopsies. Of these, Vα10, Vα11, Vα13 and Vα14 showed significantly lower frequency of expression even after correction for the number of families typed (Table II). None of the TCR families was expressed more frequently in TILs than in control biopsies.

### *TCR Vβ gene expression in TILs and control biopsies*

Twenty-one Vβ gene repertoires were analysed. The average number of Vβ genes used was 18.6 ($n = 18$) in control biop-

**Table II** Summary of TCR Vα and Vβ gene expression in NPC and control biopsies

| *TCR* | *NPC⁺ biopsies* | *Frequency* | *NPC⁻ biopsies* | *Frequency* | P | $P_c$ | *RR* | *95% CL* |
|---|---|---|---|---|---|---|---|---|
| *Vα* | +(n = *17*) | | +(n = *18*) | | | | | |
| Vα1 | 14 | 0.824 | 18 | 1.000 | | | | |
| Vα2 | 10 | 0.589 | 17 | 0.944 | 0.015 | NS | | |
| Vα3 | 10 | 0.589 | 18 | 1.000 | 0.003 | NS | | |
| Vα4 | 11 | 0.647 | 13 | 0.722 | | | | |
| Vα5 | 4 | 0.235 | 8 | 0.444 | | | | |
| Vα6 | 13 | 0.765 | 18 | 1.000 | | | | |
| Vα7 | 13 | 0.765 | 18 | 1.000 | | | | |
| Vα8 | 7 | 0.412 | 9 | 0.500 | | | | |
| Vα9 | 8 | 0.471 | 16 | 0.889 | 0.009 | NS | | |
| Vα10 | 8 | 0.471 | 17 | 0.944 | 0.002 | 0.04 | 0.05 | 0.01–0.49 |
| Vα11 | 6 | 0.353 | 16 | 0.889 | 0.001 | 0.02 | 0.07 | 0.01–0.40 |
| Vα12 | 14 | 0.824 | 18 | 1.000 | | | | |
| Vα13 | 7 | 0.412 | 18 | 1.000 | $1.1 \times 10^{-4}$ | 0.002 | 0 | |
| Vα14 | 8 | 0.471 | 17 | 0.944 | 0.002 | 0.04 | 0.05 | 0.01–0.49 |
| Vα15 | 10 | 0.588 | 17 | 0.944 | 0.015 | NS | | |
| Vα16 | 7 | 0.412 | 13 | 0.722 | | | | |
| Vα17 | 8 | 0.471 | 13 | 0.722 | | | | |
| Vα18 | 5 | 0.294 | 12 | 0.667 | | | | |
| *Vβ* | +(n = *19*) | | +(n = *18*) | | | | | |
| Vβ1 | 19 | 1.000 | 18 | 1.000 | | | | |
| Vβ2 | 19 | 1.000 | 18 | 1.000 | | | | |
| Vβ3 | 16 | 0.842 | 18 | 1.000 | | | | |
| Vβ4 | 18 | 0.947 | 18 | 1.000 | | | | |
| Vβ5.1 | 17 | 0.895 | 17 | 0.944 | | | | |
| Vβ5.2 | 17 | 0.895 | 18 | 1.000 | | | | |
| Vβ6 | 19 | 1./000 | 18 | 1.000 | | | | |
| Vβ7 | 19 | 1.000 | 18 | 1.000 | | | | |
| Vβ8 | 13 | 0.684 | 17 | 0.944 | | | | |
| Vβ9 | 16 | 0.842 | 18 | 1.000 | | | | |
| Vβ10 | 13 | 0.684 | 17 | 0.944 | | | | |
| Vβ11 | 8 | 0.421 | 16 | 0.889 | 0.003 | NS | | |
| Vβ12 | 16 | 0.842 | 17 | 0.944 | | | | |
| Vβ13 | 15 | 0.789 | 18 | 1.000 | | | | |
| Vβ14 | 4 | 0.211 | 16 | 0.889 | $3.7 \times 10^{-5}$ | 0.001 | 0.03 | 0.01–0.2 |
| Vβ15 | 13 | 0.684 | 18 | 1.000 | 0.01 | NS | | |
| Vβ16 | 0 | 0.000 | 0 | 0.000 | | | | |
| Vβ17 | 14 | 0.737 | 18 | 1.000 | | | | |
| Vβ18 | 13 | 0.684 | 17 | 0.944 | | | | |
| Vβ19 | 15 | 0.789 | 18 | 1.000 | | | | |
| Vβ20 | 11 | 0.579 | 18 | 1.000 | 0.002 | 0.04 | 0 | |

sies and 15.5 ($n = 19$) in TILs. The usage of TCR Vβ genes was more heterogeneous and most of the samples expressed most of the Vβ genes (Figure 2). However, Vβ11, Vβ14, Vβ15 and Vβ20 in TILs showed lower frequency of use than controls (Table II); Vβ14 and Vβ20 remained significant after correction.

**Figure 2** Examples of TCR Vβ gene usage in control biopsies (**a**, gel; **b**, Southern blot) and in NPC biopsies (**c**, gel; **d**, Southern blot).

*TCR gene expression in NPC PBLs and control PBLs*

To investigate the expression of TCR genes in NPC PBLs, mRNA was isolated from 15 NPC PBLs and eight control PBLs, cDNA transcribed and PCR performed. The average number of TCR Vα genes used was 10.1 in NPC PBLs and 15.3 in control PBLs. The frequency of expression of Vα2, Vα8, Vα11, Vα13, Vα14, Vα16 and Vα17 in PBLs was lower than in control PBLs, and none remained significant after correction (Table III). The average number of Vβ genes used was 17.3 in NPC PBLs ($n = 15$) and 19.3 in control PBLs ($n = 8$). The frequency of Vβ14 was again significantly lower in NPC PBLs than that in control PBLs and remained so after correction (Table III; $P_c = 0.05$; RR = 0).

*Comparison of TCR gene expression in TILs and PBLs of NPC patients*

TCR Vα genes of NPC TILs and PBLs were compared and the results showed similar average numbers of Vα use. Most of the Vα genes that were expressed in PBLs were also expressed in TILs, and most of those which were not expressed in PBLs were not expressed in TILs as well. The frequency of expression of Vα3, Vα9, Vα11 and Vα15 was lower and the frequency of Vα4, Vα16 and Vα17 was higher in TILs than in PBLs. However, the difference in frequency of expression was only significant for Vα17 ($P = 0.05$). The average Vβ gene expression in TILs and in PBLs in NPC patients was identical. The frequency of expression of Vβ10,

**Table III** Summary of TCR Vα and Vβ gene expression in PBLs and control

| *TCR* | *NPC PBLs* | *Frequency* | *Control PBLs* | *Frequency* | P | $P_c$ | *RR* | *95% CL* |
|---|---|---|---|---|---|---|---|---|
| Vα | +(n = *14*) | | +(n = *8*) | | | | | |
| Vα1 | 11 | 0.786 | 8 | 1.000 | | | | |
| Vα2 | 8 | 0.571 | 8 | 1.000 | | | | |
| Vα3 | 12 | 0.857 | 8 | 1.000 | | | | |
| Vα4 | 6 | 0.429 | 3 | 0.375 | | | | |
| Vα5 | 4 | 0.286 | 1 | 0.125 | | | | |
| Vα6 | 12 | 0.857 | 8 | 1.000 | | | | |
| Vα7 | 9 | 0.643 | 8 | 1.000 | | | | |
| Vα8 | 6 | 0.429 | 8 | 1.000 | 0.009 | NS | | |
| Vα9 | 10 | 0.714 | 8 | 1.000 | | | | |
| Vα10 | 9 | 0.643 | 8 | 1.000 | | | | |
| Vα11 | 8 | 0.571 | 8 | 1.000 | | | | |
| Vα12 | 10 | 0.714 | 8 | 1.000 | | | | |
| Vα13 | 8 | 0.571 | 8 | 1.000 | | | | |
| Vα14 | 7 | 0.500 | 8 | 1.000 | 0.02 | NS | | |
| Vα15 | 12 | 0.857 | 8 | 1.000 | | | | |
| Vα16 | 4 | 0.286 | 6 | 0.750 | | | | |
| Vα17 | 2 | 0.143 | 6 | 0.750 | 0.008 | NS | | |
| Vα18 | 4 | 0.286 | 2 | 0.250 | | | | |
| Vβ | +(n = *15*) | | +(n = *8*) | | | | | |
| Vβ1 | 15 | 1.000 | 8 | 1.000 | | | | |
| Vβ2 | 15 | 1.000 | 8 | 1.000 | | | | |
| Vβ3 | 15 | 1.000 | 8 | 1.000 | | | | |
| Vβ4 | 15 | 1.000 | 8 | 1.000 | | | | |
| Vβ5.1 | 15 | 1.000 | 8 | 1.000 | | | | |
| Vβ5.2 | 14 | 0.933 | 8 | 1.000 | | | | |
| Vβ6 | 15 | 1.000 | 8 | 1.000 | | | | |
| Vβ7 | 15 | 1.000 | 8 | 1.000 | | | | |
| Vβ8 | 11 | 0.733 | 8 | 1.000 | | | | |
| Vβ9 | 14 | 0.933 | 8 | 1.000 | | | | |
| Vβ10 | 12 | 0.800 | 5 | 0.625 | | | | |
| Vβ11 | 10 | 0.667 | 5 | 0.625 | | | | |
| Vβ12 | 13 | 0.867 | 8 | 1.000 | | | | |
| Vβ13 | 13 | 0.867 | 8 | 1.000 | | | | |
| Vβ14 | 5 | 0.333 | 8 | 1.000 | 0.003 | 0.05 | 0 | |
| Vβ15 | 15 | 1.000 | 8 | 1.000 | | | | |
| Vβ16 | 0 | 0.000 | 0 | 0.000 | | | | |
| Vβ17 | 13 | 0.867 | 8 | 1.000 | | | | |
| Vβ18 | 11 | 0.733 | 8 | 1.000 | | | | |
| Vβ19 | 13 | 0.867 | 8 | 1.000 | | | | |
| Vβ20 | 10 | 0.667 | 8 | 1.000 | | | | |

Vβ11 and Vβ15 was lower in TILs than in PBLs. The difference in Vβ15 reached statistical significance ($P = 0.02$).

### TCR and HLA

NPC in the Chinese is associated with HLA-B46 and HLA-B58 (Chan *et al.*, 1983). Vα17 was observed in 2/8 (25%) $B46^+$ NPC patients compared with 6/6 (100%) $B46^+$ controls ($P = 0.009$; RR = 0). Among the HLA-$B46^-$ subjects, the frequency of Vα17 showed no significant difference between NPC patients and controls. Similarly, Vα18 was observed in 3/8 (37.5%) HLA-$B46^+$ NPC patients compared with 5/5 (100%) $B46^+$ controls ($P = 0.044$; RR = 0). The frequency of Vα18 also showed no difference between $B46^-$ patients and controls. However, among $B46^-$ controls the frequency of Vα18 was lower (1/5, 20%) than in $B46^+$ controls
(6/6, 100%; $P = 0.015$).

### Discussion

T cells that recognise and respond to a specific antigenic peptide by activation and proliferation are considered to be clonally restricted and to express a limited number of TCR genes (Ioannides and Whiteside, 1993). The complexity of TCR use in the anti-tumour response may result from the involvement of multiple α- and β-chain regions in response to a single antigenic determinant or may reflect multiple antigenic determinants expressed on tumour cells.

Our previous study on restriction fragment length polymorphism (RFLP) of TCR genes indicated that there is polymorphism of TCR genes in NPC patients (Chen and Chan, 1994). In the present study, TCR gene expression in TILs and PBLs from NPC patients and in lymphocytes from control biopsies and control PBLs were investigated. TCR Vα genes in NPC TILs showed limited heterogeneity compared with lymphocytes from control biopsies. The frequencies of Vα2, Vα3, Vα9, Vα10, Vα11, Vα13, Vα14 and Vα15 were lower in TILs and, in particular, the frequencies of Vα10, Vα11, Vα13 and Vα14 were significantly lower than in controls even after correction for the number of Vα gene families studied. Since we were looking for the expression of many Vα and Vβ families in the same cDNA sample, differences in frequencies between patients and controls may occur by pure chance. We have attempted to correct for this by multiplying the $P$-value by the number of Vα or Vβ families typed, a common practice used in HLA and disease statistics. The frequency of Vβ gene expression in TILs was more heterogeneous than that of Vα genes. The frequencies of Vβ11, Vβ14, Vβ15 and Vβ20 were reduced compared with controls, but only Vβ14 and Vβ20 showed significantly reduced frequency after correction. In our previous study of genomic TCR patterns, NPC patients had a Vβ11/*Bam*HI allelic pattern significantly different from controls (Chen and Chan, 1994). Vβ11 was again affected in the present study, showing a lower frequency of rearrangement in the TILs, and the two findings may be related.

TILs showed individual variation in TCR expression. The differences in TCR gene expression detected among the individuals may be related to TCR V-gene polymorphisms that impose the utilisation of different V-gene segments for the recognition of the same antigen. Restriction of TCR use may also be influenced by a person's HLA type. In this regard, it is interesting to note that NPC patients with HLA-B46 had lower frequencies of Vα17 and Vα18 than to $B46^+$ controls, suggesting that the combination of HLA restriction and lack of TCR gene use may be important in the pathogenesis of NPC. Lymphocytes from biopsies of all normal controls with HLA-B46 expressed Vα17 and Vα18, but only 25% and 37.5% of TILs, respectively, expressed these markers.

TILs in NPC are mainly mature T lymphocytes with different ratios of $CD4^+/CD8^+$ cells. It is important to investigate the TCR use in T-cell subgroups. We have preliminarily analysed TCR use between total and $CD4^+$ T cells in the same patients and controls. While there was no difference in TCR use between total T cells and $CD4^+$ T cells in the two controls, both NPC patients showed even more restricted TCR family use in the $CD4^+$ T cells compared with total T cells. The number of patients and controls studied was too small to make any firm conclusion but suggests that TCR use in NPC may be even more restricted in the T-helper cell subpopulation.

The present results showed that Vα10, Vα11, Vα13, Vα14, Vβ14 and Vβ20 were underexpressed in NPC TILs, suggesting that these genes may have been deleted. Nasopharyngeal biopsies are usually small, and it may be pure chance that T cells with certain TCR rearrangements are not represented. However, arguing against this possibility was the finding that, in general, those TCR rearrangements not present in TILs were also not represented in the peripheral blood of most patients. Not all patients or controls have matching biopsies and peripheral blood samples. In those that have matching pairs, the concordance of Vα and Vβ bands between biopsy and peripheral blood was usually over 90% (e.g. Vα10, 91%; Vα13, 91%; Vβ14, 92%). TILs and PBLs in our study had been cultured *in vitro* for 1–2 weeks, and there was no difference in culture time between NPC patients and controls. The cultures were all growing well at the time of harvest. It is possible that only certain TCR families were selected for expansion. However, in our study, no preferential use of certain TCR genes was observed, and there is so far no evidence to support this. TCR Vα gene expression with or without culture showed little difference in other studies (Davies *et al.*, 1991), and culture with IL-2 reduced rather than enhanced the degree of restriction. It is possible that T cells with certain TCRs fail to grow in the presence of IL-2. However, T cells with these TCRs grew well in control cultures. On the other hand, immunosuppressed T cells may behave differently, and it is possible that suppressed T cells with certain TCRs may not grow in the presence of IL-2, and we are exploring this possibility. Whether the deletion of these TCR genes leads to failure of T-cell recognition of tumour peptide/HLA complexes resulting in the escape of NPC tumours from immune surveillance, or whether NPC results in the deletion of specific TCR genes also need to be investigated. We found no specific TCR gene use in NPC, but rather a specific deletion of certain TCR genes, and this finding together with HLA restriction may be important in the pathogenesis of NPC.

### Abbreviations

TIL, tumour-infiltrating lymphocyte; PBL, peripheral blood lymphocyte; NPC, nasopharyngeal carcinoma; HLA, human leucocyte antigen; TCR, T-cell receptor; RPMI, Roswell Park Memorial Institute; IL-2, interleukin 2; PBS, phosphate-buffered saline.

### Acknowledgements

The authors wish to thank the technical staff of the WHO Immunology Centre for their excellent technical assistance. This study is supported by National University of Singapore Grant No. 910416. Y Chen is supported by a Research scholarship from the National University of Singapore.

### References

BENNETT WT, PANDOLFI F, GROVE BH, HAWES GE, BOYLE LA, KRADIN RL AND KURNICK JT. (1992). Dominant rearrangements among human tumor infiltrating lymphocytes. *Cancer*, **69**, 2379-2384.

BEN-NUN A, LIBLAU RS, COHEN L, LEHMANN D, TOURNIER-LASSERVE E, ROSENZWEIG A, ZHANG JW, RAUS JCM AND BACH MA. (1991). Restricted T cell receptor Vβ gene usage by myelin basic protein-specific T cell clones in multiple schlerosis: predominant genes vary in individuals. *Proc. Natl Acad. Sci. USA*, **88**, 2466-2470.

CHAN SH, DAY NE, KUNARATNAM N, CHIA KB AND SIMONS MJ. (1983). HLA and nasopharyngeal carcinoma in Chinese-a further study. *Int. J. Cancer*, **32**, 171-176.

CHAN SH, LIN YN, WEE GB, KOH WH AND BOEY ML. (1994). HLA class 2 genes in Singaporean Chinese rheumatoid arthritis. *Br. J. Rheumatol.*, **33**, 713-717.

CHAN SH, TAN CB, LIN YN, WEE GB, DEGLI-ESPOSTI MA AND DAWKINS RL. (1993). HLA and Singaporean Chinese myasthenia gravis. *Int. Arch. Allergy Immunol.*, **101**, 119-125.

CHAN SH, YEO PPB, LUI KF, WEE GB, LIM P AND CHEAH JS. (1978). HLA and thyrotoxicosis (Graves' disease). *Tissue Antigen*, **13**, 73-74.

CHAN SH, FENG PH, SPRINIVASAN N, WEE GB AND CHAN HC. (1981). HLA and systemic lupus erythematosus in Chinese. *Hum. Immunol.*, **3**, 345-350.

CHAN SH, CHEW TS AND KUNARATNAM N. (1979). Cell mediated immunity to EBV in nasopharyngeal carcinoma. *Lancet*, **i**, 884-885.

CHEN Y AND CHAN SH. (1994). Polymorphism of T-cell receptor genes in nasopharyngeal carcinoma. *Int. J. Cancer*, **56**, 830-833.

DAVIES TF, MARTIN A, CONCEPCION ES, GRAVES P, COHEN L AND BEN-NUN A. (1991). Evidence of limited variability of antigen receptors on intrathyroidal T cells in autoimmune thyroid disease. *New Engl. J. Med.*, **325**, 238-244.

DAVIES TF, MARTIN A, CONCEPCION ES, GRAVES P, LAHAT N, COHEN WL AND BEN-NUN A. (1992). Evidence for selective accumulation of intrathyroidal T lymphocytes in human autoimmune thyroid disease based on T cell receptor V gene usage. *J. Clin. Invest.*, **89**, 157-162.

GIEGERICH G, PETTE M, MEINL E, EPPLEN JT, WEKERLE H AND HINKKANEN A. (1992). Diversity of T cell receptor α and β chain genes expressed by human T cells specific for similar myelin basic protein peptide/major histocompatibility complexes. *Eur. J. Immunol.*, **22**, 753-758.

IOANNIDES CG AND WHITESIDE TL. (1993). T cell recognition of human tumors: implications for molecular immunotherapy of cancer. *Clin. Immunol. Immunopathol.*, **66**, 91-106.

LEE BW, CHAN SH, TAN SH, WONG HB AND TAN CL. (1983). HLA system in Chinese children with insulin-dependent diabetes mellitus. *Austr. Paediatr. J.*, **19**, 34-35.

MARTIN R, UTZ U, COLIGAN JE, RICHERT JR, FLERLAGE M, ROBINSON E, STONE R, BIDDISON WE, MCFARLIN DE AND MCFARLAND HF. (1992). Diversity in fine specificity and T cell receptor usage of the human CD4+ cytotoxic T cell response specific for the immunodominant myelin basic protein peptide 87-106. *J. Immunol.*, **148**, 1359-1366.

MOSS DJ, CHAN SH, BURROWS SR, CHEW TS, KANE RG, STAPLES JA AND KUNARATNAM N. (1983). Epstein-Barr virus specific T cell response in nasopharyngeal carcinoma patients. *Int. J. Cancer*, **32**, 301-305.

NITTA T, OKSENBERG JR, RAO NA AND STEINMAN L. (1990). Predominant expression of T cell receptor Vα7 in tumor infiltrating lymphocytes of uveal melanoma. *Science*, **249**, 672-674.

NITTA T, SATO K, OKUMURA K AND STEINMAN L. (1991*a*). An analysis of T cell receptor variable region genes in tumor infiltrating lymphocytes within malignant tumors. *Int. J. Cancer*, **49**, 545-550.

NITTA T, BELL R, OKUMURA K, SATO K AND STEINMAN L. (1991*b*). T cell receptor Vβ gene expression differs in tumor infiltrating lymphocytes within primary and metastatic melanoma. *Cancer Res.*, **51**, 5565-5569.

OKSENBERG JR, STUART S, BEGOVITCH AB, BELL RB, ERLICH HA, STEINMAN L AND BERNARD CCA. (1990). Limited heterogeneity of rearranged T cell receptor Vα transcripts in brains of multiple sclerosis patients. *Nature*, **345**, 344-346.

SHANMUGARATNAM K, CHAN SH, DE-THE G, GOH EH, KHOR TH, SIMONS MJ AND TYE CY. (1979). Histopathology of nasopharyngeal carcinoma. Correlations with epidemiology, survival rates and other biological characteristics. *Cancer*, **44**, 1029-1044.

SIOUD M, KJELDSEN-KRAGH J, QUAYLE AJ, WIKER HG, SORSKAAR D, NATVIG JB AND FORRE O. (1991). Immune responses to 18.6 and 30-kDa mycobacterial antigens in rheumatoid patients, and Vβ usage by specific synovial T cell lines and fresh T cells. *Scand. J. Immunol.*, **34**, 803-812.

SIOUD M, KJELDSEN-KRAGH J, SULEYMAN S, VINJE O, NATVIG JB AND FORRE. (1992). Limited heterogeneity of T cell receptor variable region gene usage in juvenile rheumatoid arthritis synovial T cells. *Eur. J. Immunol*, **22**, 2413-2418.

SOTTINI A, IMBERTI L, GORLA R, CATTANEO R AND PRIMI D. (1991). Restricted expression of T cell receptor Vβ but not Vα genes in rheumatoid arthritis. *Eur. J. Immunol.*, **21**, 461-466.

SOUTHERN EM. (1975). Detection of specific sequences among DNA fragments separated by gel electrophoresis. *J. Mol. Biol.*, **98**, 503-517.

SUMIDA T, YONAHA F, MAEDA T, TANABE E, KOIKE T, TOMIOKA H AND YOSHIDA S. (1992). T cell receptor repertoire of infiltrating T cells in lips of Sjögren's syndrome patients. *J. Clin. Invest.*, **89**, 681-685.

UTZ U, BIDDISON WE, MCFARLAND HF, MCFARLIN DE, FLERLAGE M AND MARTIN R. (1993). Skewed T cell receptor repertoire in genetically identical twins correlates with multiple sclerosis. *Nature*, **364**, 243-247.

WEIDMANN E, ELDER EM, TRUCCO M, LOTZE MT AND WHITESIDE TL, (1993). Usage of T cell receptor Vβ chain genes in fresh and cultured tumor infiltrating lymphocytes from human melanoma. *Int. J. Cancer*, **54**, 383-390.

WUCHERPFENNIG KW, NEWCOMBE J, LI H, KEDDY C, CUZNER ML AND HAFLER DA. (1992). T cell receptor Vα-Vβ repertoire and cytokine gene expression in active multiple sclerosis lesions. *J. Exp. Med.*, **175**, 993-1002.

WUCHERPFENNIG KW, OTA K, ENDO N, SEIDMAN JG, ROSENZWEIG A, WEINER HL AND HAFLER DA. (1990). Shared human T cell receptor Vβ usage to immunodominant regions of myelin basic protein. *Science*, **248**, 1016-1019.